# Empowerment, Communication, and Navigating Care: The Experience of Persons With Spinal Cord Injury From Acute Hospitalization to Inpatient Rehabilitation

**DOI:** 10.3389/fresc.2022.904716

**Published:** 2022-05-31

**Authors:** Jacqueline A. Krysa, Marianne Pearl Gregorio, Kiran Pohar Manhas, Rob MacIsaac, Elizabeth Papathanassoglou, Chester H. Ho

**Affiliations:** ^1^Neurosciences, Rehabilitation, and Vision Strategic Clinical Network, Alberta Health Services, Edmonton, AB, Canada; ^2^Division of Physical Medicine and Rehabilitation, University of Alberta, Edmonton, AB, Canada; ^3^Faculty of Nursing, University of Alberta, Edmonton, AB, Canada; ^4^Community Health Sciences, Cumming School of Medicine, University of Calgary, Calgary, AB, Canada; ^5^Spinal Cord Injury Alberta, Edmonton, AB, Canada

**Keywords:** Spinal Cord Injury (SCI), patient experience, rehabilitation, acute care, inpatient rehabilitation

## Abstract

**Background:**

Spinal cord injury (SCI) results in long-term functional impairments that significantly impact participation and role in the community. Newly injured persons are often reintroduced to the community with significant deficits in knowledge, including how to access and navigate community resources and supports. This warrants a better understanding of the patient experience of in-hospital care and discharge planning to ensure individuals with SCI are best supported during transitions in care and while living in the community.

**Objective:**

To explore the lived experience of persons with acute SCI and their perceptions of care, focusing on the initial hospital experiences to inpatient rehabilitation.

**Methods:**

A phenomenological research study was conducted using semi-structured interviews. Eligible participants had differing etiologies of SCI (including non-traumatic and traumatic SCI), were over the age of 18 at the time of initial care, and experienced acute hospital and inpatient rehabilitation at an Alberta-based institution within the last 10 years. One-on-one interviews took place between March and June 2021 over telephone or virtual platforms (Zoom). Interview transcripts, and field notes developed the text, which underwent hermeneutic analysis to develop central themes.

**Results:**

The present study included 10 participants living with an SCI in Alberta, Canada. Most participants (80%) were male. Participants' age ranged from 24 to 69 years. The median years since initial SCI was 3 years. Interviews lasted 45–75 min. Seven participants identified as having a traumatic SCI injury and three identified as having a non-traumatic SCI. The interplay between empowerment and disempowerment emerged as the core theme, permeating participants' meanings and perceptions. Three main themes emerged from the interviews regarding the perceptions of the SCI patient experience. Each theme represents a perception central to their inpatient experience: *desire to enhance functional independence* to empower confidence and self-management; *need for effective communication* with healthcare providers to support recovery; and *navigating appropriate care supports* to enhance preparedness for discharge and returning home.

**Conclusion:**

This study demonstrates the significant need to enhance education of person/family-centered SCI care, foster positive communication between care recipients and care providers, and facilitate better in-hospital access to appropriate navigation and wayfinding supports.

## Introduction

Spinal Cord Injury (SCI) is a highly disruptive and debilitating condition that interferes with sensory, motor and autonomic function, and directly impacts physical, psychological, and social wellbeing (1, 2). SCI can arise from trauma (e.g., injuries from falls), or from non-traumatic conditions (e.g., degenerative disease) (2). Recovery from SCI is divided into three main phases of care: acute, inpatient rehabilitation, and longitudinal outpatient care (3). Acute in-hospital care consists of initial post-injury care, while inpatient rehabilitation and longitudinal care delivers post-injury care in both the inpatient and outpatient setting (3). Inpatient rehabilitation for persons with SCI focuses on enhancing physical capacity and performance of activities of daily living to prepare for discharge (4). SCI is a lifelong condition and is often associated with chronic multi-morbidity, including the development of secondary health complications such as urinary tract infections and pressure ulcers (5). SCI has also been shown to impact an individual's mental health, with evidence of increased anxiety, depression, substance abuse, and overall cognitive changes (6).

SCI results in long-term functional impairments that significantly impacts participation and role in the community, such as changes to employment, and living situation (7, 8). Persons with SCI are deemed ready for discharge from hospital after they receive appropriate education on their condition, managing and preventing secondary complications at home, and strategies to participate in activities of daily living based on their neurological level of injury (9). However, newly-injured persons are often reintroduced to the community with significant deficits in knowledge, including how to access and navigate community resources and supports (10). SCI patient education is fundamental to supporting a seamless transition from hospital to community and living with a disability (11). This emphasizes the need to focus on long-term rehabilitation goals, including community re-integration, during the acute and subacute phases of SCI care (12). It also warrants a better understanding of the patient experience of in-hospital care and discharge planning. These insights may better identify the interplay of factors that support transitions from hospital to home (13).

Most studies exploring the patient experience of SCI in-hospital care focus on inpatient rehabilitation (14–16). A meta-synthesis of qualitative evidence concerning the patient experience of rehabilitation following SCI concluded that rehabilitation care services must be informed by the experiences and perspectives of people with SCI to ensure care delivery is appropriate and effective (14). SCI patient experience literature has also addressed participation in decision-making (17–19). These studies found many barriers to collaborative decision-making, including professional paternalism as well as physical, psychological, and environmental factors (17–19). Considering the complexity of the SCI care, a clearer understanding of the lived experience of the healthcare system, specifically acute hospital care and inpatient rehabilitation, following an SCI is essential to informing the delivery of care and appropriate resources to support patient readiness for discharge and reintegration into the community.

## Materials and Methods

### Design

This study was phenomenological and involved semi-structured interviews. A phenomenological method was used to remain open to participants' narratives and meaning-making while investigating their lived experience, as Phenomenology aims to reveal meaning behind the subjective experiences of individuals and groups (20). The research team used a post-positivism epistemological stance, which acknowledges that the research findings are bound by context, and considers both researcher and theoretical biases in the design and interpretation of the results (21). The COREQ checklist used as a guideline to ensure accurate reporting throughout the manuscript (22).

### Sampling

Purposive sampling for maximum variation helped capture diverse experiences of persons living with SCI in Alberta, Canada (i.e., males and females, persons from rural and urban communities, different levels and etiologies of SCI). This study aimed to recruit 8–15 individuals living with an SCI.

### Eligibility Criteria

Study inclusion criteria included: individuals over the age of 18 at the time of initial care; and experience of acute in-hospital care and inpatient rehabilitation on neuro-trauma units for their SCI at an Alberta-based institution within the last 10 years. The time limit was set to balance a minimization of recall bias with improved feasibility in recruitment. Participants with different levels and etiology of SCI (including non-traumatic and traumatic SCI) were eligible for participation. Participants were excluded if they were unable to give informed consent by themselves or with assistance from a close relation.

### Recruitment

We partnered with a community SCI agency (Spinal Cord Injury (SCI) Alberta) based in Alberta, Canada to support participant recruitment. SCI Alberta offers client support, family and peer supports, as well as community services for persons living with SCI across the province. The provincial reach and contacts associated with SCI Alberta enabled widespread engagement and advertising for recruitment to advance diversity of representation. SCI Alberta recruited eligible participants by advertising the study on their social media page or by directly contacting eligible individuals by phone to share details of the study. If recruited by social media, interested participants directly contacted the research team. For those recruited directly, SCI Alberta obtained verbal consent from interested participants to provide their name and contact information to the research team. The research team then contacted participants by email or phone to review informed consent and organize study participation.

### Data Collection

A semi-structured interview guide with open-ended questions was developed based on relevant SCI patient experience literature (23–26), as well as discussions with subject matter experts, including three persons with lived experience of SCI. The interview questions covered the experience of injury and initial care, expectations of care, and other considerations of care including emotional or psychological care, support for family and care providers, role in decision making, and transitions in care. The full interview guide is available (see Supplementary File S1). Interviews were conducted one-on-one by phone or virtual platform (Zoom). Interviews took place between March and June 2021 and were performed by the lead researcher (JAK), a female post-doctoral fellow, experienced in qualitative interview analysis for health services research. The interviewer connected with all participants prior to the interview to explain the purpose of the study, review the consent form, organize the interview, and answer any questions. Fourteen individuals were contacted about the study and 10 were interested in participating. All interviews were audio-recorded, and confidentially transcribed verbatim. All participants were assigned a study ID and any potentially-identifiable details were removed from the transcripts.

### Data Analysis

Two researchers independently reviewed all interview transcripts and field notes (JAK, MPG). Qualitative analysis was conducted using NVIVO-12 software (QSR International 2022). To ensure consistency of data analysis, interviews were analyzed according to Dieklemann's 7-step hermeneutic analysis to ensure research validity and credibility (27). A hermeneutic cycle was used to determine a valid and consistent understanding of the participant's experience of the phenomena, and uncover deeper meaning and emerging themes from the text (28, 29). Overall understanding of the lived experience was ensured by co-designing and reviewing the interview guide with three persons with lived experience of SCI as well as employees of SCI Alberta. Interview transcripts and field notes enabled in-depth description of data. Field notes were written to supplement the data and captured the interviewer's initial impressions and participant non-verbal cutes (when participants had their video turned on). Two transcripts were independently coded by four authors (JAK, MPG, KPM, EP) into meaning-bearing units (codes) related to the study objectives. Any disagreements on interpretation were resolved by revisiting the original interview transcripts. The codes were compared and discussed until agreement was achieved by all researchers. This subset analysis built a set of defined codes that were applied to subsequent interviews. The codes generated were refined and expanded as additional interviews were analyzed. The process of interviewing and analysis was iterative. The connections between the identified themes were examined to explore relationships or causality, and to gain a more in-depth understanding of the participant's experience. All members of the research team reviewed the draft themes and exemplar quotes prior to subsequent analysis of interpretation.

### Ethical Considerations

This study was approved by the University of Alberta Research Ethics Board (Pro00105843). All participants were informed about the study and consented to participating prior to the interview.

## Results

### Characteristics of Study Participants

**Figure 1:**
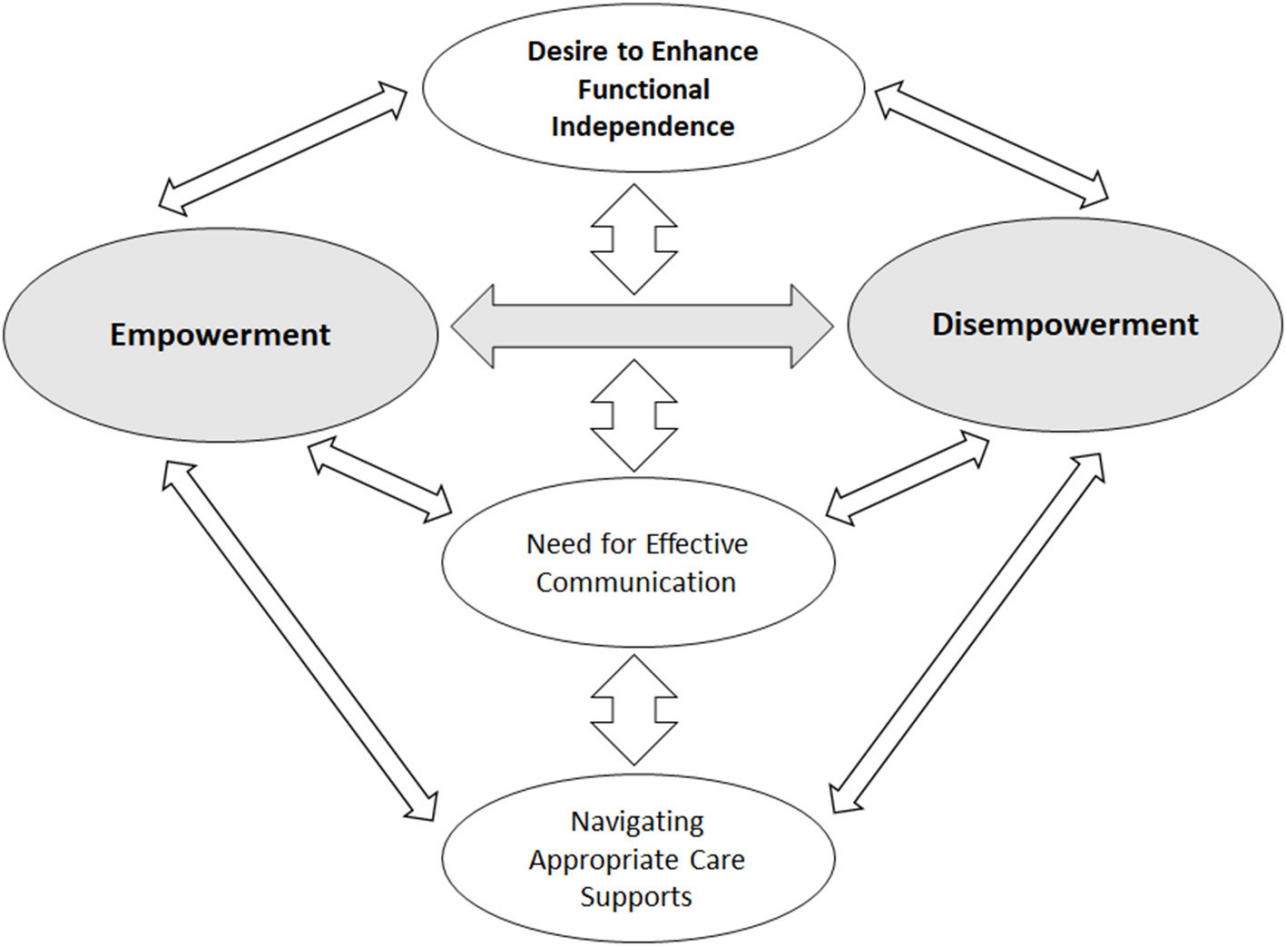
Thematic framework underlying the experience of persons with SCI regarding in-hospital care.

The study interviewed 10 participants living with an SCI in Alberta, Canada between March and June 2021. Interviews lasted 45–75 min. Participants were recruited until richness and data saturation were achieved, whereby additional interviews brought no additional insights to the primary research question (30), and data saturation was determined by the interviewer and confirmed by a second researcher (MPG) involved in transcript analysis. Participant demographics (age, sex, level of injury, years since SCI, and hospital facility visited) were self-reported during the interview. Characteristics of study participants are reported in Table 1. The majority of participants (80%) were male. Participants' age ranged from 24 to 69 years. The median years since initial SCI was 3 years (range 2–9 years). All participants were interviewed after discharge from hospital or inpatient rehabilitation. During their initial care experience, six participants were living in urban regions and four were living in rural regions of the province. There were seven participants that self-reported a traumatic SCI injury and three that self-reported a non-traumatic SCI. Participants discussed care experiences from two acute hospitals and two inpatient rehabilitation care sites across Alberta. There were three participants that received initial acute care outside of the province prior to admission to an Albertan inpatient rehabilitation facility. Thematic coding led to the development of a conceptual representation of the in-hospital care experiences for persons with SCI in Alberta (Figure 1, Table 2).

**Table 1:** Participant characteristics.

**Participant**	**Sex**	**Age**	**SCI**	**Years**	**Geographical**
			**etiology**	**since**	**residence**
				**SCI**	**(urban vs**.
					**non-urban)**
1	Male	27	Traumatic	2	Urban
2	Male	57	Traumatic	4	Urban
3	Male	43	Non-traumatic	2	Urban
4	Female	39	Traumatic	9	Urban
5	Male	34	Traumatic	2	Non-urban
6	Female	44	Traumatic	2	Urban
7	Male	64	Non-traumatic	4	Urban
8	Male	24	Traumatic	6	Non-urban
9	Male	64	Non-traumatic	3	Non-urban
10	Male	69	Traumatic	2	Non-urban

**Table 2:** Thematic framework underlying the SCI patient experience of in-hospital care.

**Themes**	**Sub-Themes**
Desire to enhance functional independence	• Self-advocacy • Expecations of care • Access to specialized care and education
Need for effective communication	• Empathy and optimism • Shared decision-making • Motivation from staff and peers
Navigating appropriate supports	• Community resources to enhance functional recovery • Peer, family, and caregiver support • Navigation support from health care providers

### In-hospital Care Experiences of Persons With SCI

The *interplay between empowerment and disempowerment* emerged as the core theme, permeating participants' meanings and perceptions. Participants felt the need to be self-empowered for recovery amidst conditions that often deprived them of control over their situation. Three main themes emerged from the patient experience: (1) *desire to enhance functional independence;* (2) n*eed for effective communication*; (3) *navigating appropriate care supports*. Each theme represents a perception central to their inpatient experience across three sub-themes (Table 2).

These themes are discussed in further detail below. Additional exemplar quotes are provided in Table 3.

**Table 3:** Representative quotes.

**The interplay between empowerment and disempowerment**	
“*My goal was to be able to do my own in and out [catheterization], but I wasn't able to do it. If I wanted to be trained, it was up to me to ask one of the nurses to teach me. There wasn't anything formally set up…”* (Participant #6, female, 2 years post-SCI) “*Nobody tells you that it's gonna be okay when you get out. That you're gonna be fine. They just tell you no, you're never gonna walk again…and then when you get out of the room and you see other people, you realize, well things do change in a couple of years.”* (Participant #2, male, 4 years post-SCI)	
**Desire to enhance functional independence**	
Self-advocacy	“*I spent most of my time doing my own research whenever I could. My husband [and I]…pushed as hard as we could to get more therapy and productive therapy…I need[ed] to be able to transfer. I need[ed] to be independent”* (Participant #6, female, 2 years post-SCI)
	“*I did a lot [of research] on my own. I really think that they could learn about so many outside programs…I managed to get myself in the system but I did it all on my own*.” (Participant #2, male, 4 years post-SCI)
Expectations of care	“*At [the inpatient rehabilitation facility]…they worked a little bit with me, [but] not as much as I thought they would*.”* (*Participant #10, male, 2 years post-SCI*)*
	“*The amount of time and resources [the hospital] had to cover an initial physiotherapy session was not enough. I stayed 2 weeks there without starting anything. It was just once a week I think or twice a week for 15 to 20 minutes”* (Participant #1, male, 2 years post-SCI)
Access to specialized training and education	“*When the [healthcare providers] spoke at [educational sessions], it was all really dated stuff. The [handouts] on the catheter changes were something that had been photocopied for the last 15 years and nothing had been updated.”* (Participant #2, male, 4 years post-SCI)
	“*Spinal cord injuries involve many aspects that are traumatizing and life changing like bladder, bowel and many other things, like infections. And it seems like the [acute care hospital] didn't really know what to do about it*.” (Participant #1, male, 2 years post-SCI)
**Need for effective communication**	
Empathy and optimism	“*I couldn't have asked for better care. The [health care aids and nurses] were always there for me. If you're there that long, you get to know them…And they give you something to talk about*.” (Participant #7, male, 4 years post-SCI)
	“*Health care professionals need to be aware that their words carry weight. And that the patients of theirs put one hundred percent of their trust in the health care professionals. And when their health care professional is not being professional [it] is putting the [patients] down*.” (Participant #4, female, 9 years post-SCI)
	“*It's almost they detach from the patient emotionally so they just treat you and they do whatever they have to do without any care*.” (Participant #1, male, 2 years post-SCI)
Shared decision making	“*We fought and argued and said ‘No, we need more rehab.' And the answer was…‘We're waiting on your discharge only because you don't have equipment at home. But once your equipment is at home then you're going to be discharged.”'* (Participant #6, female, 2 years post-SCI)
	“*For the most part, they just show up and did their thing. There's not much communication between the hospital and the patients in terms of what's happening. It's left up to the patient to like discover these things for the most part, I find.”* (Participant #5, male, 2 years post-SCI)
Motivation from staff and peers	“*The [healthcare provider] wasn't…pushing the limit on my capabilities.”* (Participant #8, male, 6 years post-SCI)
	“*It almost felt like, what they're trying to do is prepare people to go to a nursing home or something, rather than really get them going [in the community].”* (Participant #6, female, 2 years post-SCI)
**Navigating appropriate supports**	
Community resources to enhance functional recovery	“*I was going to [to the community] to do my activities because at [the rehabilitation hospital] they had an FES cycle, but never wanted me to use it*.” (Participant #1, male, 2 years post-SCI)
	“*I don't think [the hospital staff] had enough information on my community...We managed to make it work because…I knew [my home town] really well.”* (Patient #9, male, 3 years post-SCI)
Peer, family, and caregiver support	“*We had no friends or family here and my wife was worried that I wasn't seeing enough people or chatting with people and so she found [a community peer support organization] on the website and phoned in for me.”* (Participant #3, male, 2 years post-SCI)
	“*My siblings kept a book and kept notes of what was happening every day and then they read back to me …so I [was] going by what they told me*.” (Participant #10, male, 2 years post-SCI)
Navigation support from health care providers	“*They did a great job of teaching me everything that I needed to do to be prepared to like leave the hospital…because I was injured at work, I have a lot of resources for care outside of the hospital and probably even better quality than what the hospital can provide”* (Participant #5, male, 2 years post-SCI)
	“*The social worker I had, she was absolutely awesome. She gave me all the right information. We tried to apply for everything we could for having some government funding for covering basically the basic needs.”* (Participant #1, male, 2 years post-SCI)

### Core Theme: Interplay Between Empowerment and Disempowerment

A core theme underscoring the SCI perception of acute and inpatient rehabilitation was the interplay between generating empowerment from themselves, staff, peers, and family to enhance their functional recovery and the perceived disempowerment from the healthcare system to achieve this goal.

Disillusionment with the healthcare system, and obstacles to their rehabilitation created a tension between the need for empowerment in their rehabilitation journey, and an often disempowering milieu of care. Throughout their care experience, participants were generally motivated to enhance their overall functional independence, and gain as much knowledge as possible to best support themselves in the community following discharge. Their inherent motivation and trust in the healthcare system was often discouraged by a perceived lack of SCI education during acute hospital care and was accompanied by apparent demotivating personnel and limited personalized care plans during inpatient rehabilitation.

“*There is a lot of encouragement that needs to happen [during recovery]. It's understandable that you need to help people accept their injury. But to basically shut down any kind of hope - that is the worst thing…Trying isn't going to hurt us. We're going to fall and get up as many times as it takes*.” (Participant #6, female, 2 years post-SCI)

In our participants, the tension between the need to be empowered and barriers to empowerment appeared to fuel their motivation to maximize their outcomes regardless of obstacles. This was realized through active agency in discovering and utilizing diverse resources in the community, peer-groups, SCI societies and rehabilitation centers with the help of their families. Ultimately, participants discovered a path to empowerment through claiming agency of their rehabilitation and recovery.

“*When I was in the [inpatient rehabilitation facility], there was nobody there to tell you about [community] programs and funding…That is really a shame because we find out about it all on our own after the fact. I believe [the health care providers] should be handing out information like that to help people realize that when you get out, there are supports for you.”* (Participant #2, male, 4 years post-SCI)

### Desire to Enhance Functional Independence

All participants expressed a desire to increase their knowledge of SCI and rehabilitation strategies to enhance their overall independence and preparedness for discharge. Participants were motivated to obtain the necessary skills to increase their functional capacity and independence over time. Many participants expressed a strong desire to learn care practices that better enabled them to manage their own care. Some participants felt that they had to be assertive or go out of their way to receive appropriate information and resources to support their recovery. This was especially noted during the acute, in-hopsital phase of care.

“*I researched it all on my own...I was very fit before…I knew that I had to get my movement back. I have to move my body to maintain whatever muscle mass I had and I knew I had to do it right to get it back*.” (Participant #4, female, 9 years post-SCI)

Unmet expectations of care was often discussed during the inpatient rehabilitation phase of care and represented a common negative experience and barrier toward participant goal attainment. Participants generally expected greater access to one-on-one physical-therapy prior to admission to inpatient rehabilitation. They felt that the care received during their stay in inpatient rehabilitation was not enough to enhance their overall functioning and preparedness for discharge.

“*I was expecting when I went to the [Inpatient Rehabilitation Facility] that it was going to be full on rehabilitation. But when I got there I…was scheduled for 1 h of physiotherapy, 1 h for occupational therapy, and then…the rest of the time you're basically in your bed.”* (Participant #2, male, 4 years post-SCI)

There were mixed feelings on the specialized training and types of rehabilitation programs offered in the inpatient rehabilitation setting. While some participants found benefit from the standardized rehabilitation programs, many felt that more personalized, patient-centered rehabilitation opportunities would offer greater benefits.

“*Probably for a lot of people [the rehabilitation program] was helpful…but for me it was waste of time. I didn't find it helpful for me and that was very frustrating.”* (Participant #7, male, 4 years post-SCI)

When describing their experiences of acute, in-hospital care, participants often cited a lack of provider competencies in specialized SCI care, knowledge of local resources and supports, as well as differing care practices between hospital units. This perceived knowledge gap reduced participant's overall confidence in their care providers, and for some, resulted in insufficient training to support self-management following discharge.

“*The techniques they were teaching, like transferring yourself in and out of the wheelchair…weren't how they do them at the [other hospital]. You are receiving mixed information in the transition [from the] hospital setting to a rehabilitation hospital”* (Participant #5, male, 2 years post-SCI)

### Need for Effective Communication

Communication with care providers, both positive and negative, influenced participants' motivation to participate in rehabilitation and their perceived readiness for discharge. Poor communication with care providers was identified as a negative experience by most participants. Many described a general lack of empathy and optimism from acute hospital care providers when discussing their prognosis, with some citing that certain providers were discouraging or demotivating of their aspirations for rehabilitation.

“*[The staff] always tried to turn down the will I had and the energy I was putting toward rehab, to the point that I had to push against their negativity.”* (Participant #1, male, 2 years post-SCI)

Several participants described feeling excluded from decisions regarding their care and perceived this as a barrier to receiving appropriate care and feeling ready for discharge. Participants also described that certain healthcare providers at inpatient rehabilitation facilities were not motivating or challenging them enough to progress to their desired level of functional independence.

“*They have a team of professionals that make all the decisions for you. But…you're not a part of that conversation. Everything is done for you, not with you”* (Participant #4, female, 9 years post-SCI)

Many participants described building relationships with healthcare staff as a positive experience while in inpatient rehabilitation. These relationships were often described by examples of empathetic communication and encouragement from healthcare providers, which overall built trust and enhanced participant's motivation for rehabilitation and preparation for discharge.

“*The people at the [Inpatient Rehabilitation Facility] said that I was one of the hardest workers that they ever had. [That] made me feel good and just kept me in the right frame of mind…to do the things that I used to be able to do...”* (Participant #3, male, 2 years post-SCI)

### Navigating Appropriate Care Supports

Navigating or learning about and accessing appropriate rehabilitation and recovery supports, including information on community resources and financial supports, were identified as a facilitator of positive patient experience. Many participants learned of, and visited, community facilities during their hospital stay as a means to access additional rehabilitation or related resources to support their recovery. Participants often learned of these programs by themselves, a social worker, or through family and peer support persons.

“*I started going to this [community] rehabilitation center and there, I really improved my function, because they made me stand, and taught me how to [manage] my spasms”* (Participant #1, male, 2 years post-SCI)

Family and caregiver support was an important source of way-finding for participants. A family member was usually present in the hospital throughout the patient care journey. The family member would often keep track of the participant's procedures and treatments. They would search for community resources and supports to help better prepare their loved one for discharge and return home.

*Thankfully, since my mom was involved with health care as a career she knew what was expected and the majority of the resources…I feel like if I didn't have [a] mom with a health care background, it would have been a completely different situation*. (Participant #8, male, 6 years post-SCI)

Peer support volunteers were helpful at answering participant questions, setting expectations, educating about SCI care and self-management, as well as identifying local community resources and supports throughout the patient care journey.

“*[The peer support volunteer] came in once a month…She was [a] really good person to talk to if you took advantage of her knowledge… they offered a support system for questions for home care and stuff like that.”* (Participant #7, male, 4 years post-SCI)

Participants noted that communication with social workers and occupational therapists was helpful for resource finding, setting up financial supports, as well as preparing for discharge and return to home.

“*The social worker was great…I once [asked], ‘just come in and talk to me', because I was struggling for a while with minor depression, but they were good. They meant to get [me] back in the community, communicating with various government [agencies] and they were excellent and telling me what was there.”* (Participant #9, male, 3 years post-SCI)

## Discussion

This study highlights experiences of persons living with SCI during the acute hospital care and inpatient rehabilitation, from which we can derive recommendations to improve quality of care to best support persons during transitions in care and return to community. The experience of in-hospital care for persons with SCI underlines the need for empowerment to support recovery, return to home, and community re-integration. Our findings highlight a significant need to enhance education of person/family-centered SCI care; foster positive communication between care recipients and healthcare providers; and facilitate better in-hospital access to appropriate community supports. The identified themes have direct implications on aspects of community re-integration, and showcase the impact of the patient experience on this essential phase of care.

### Desire to Enhance Functional Independence

This study revealed that, during their initial hospital visit, persons with SCI have significant motivation to enhance their functional capacity to become as independent as possible. Participants had a strong desire to participate in their own care planning and self-management, but often felt they had insufficient information on their condition early in their care experience and needed to advocate for their own information or resources. A previous study on 214 patients with traumatic and non-traumatic SCI reported that, at the time of discharge, only 47% of patients reported good knowledge about SCI self-care, while 22% reported poor knowledge (31). Persons living with SCI have a high risk of secondary complications over the first year of discharge (32). A study by May and colleagues found that SCI patients who experienced care at an inpatient rehabilitation facility consistently rated bladder, bowel, and skin care as topics on which they frequently sought information (33). Early and frequent in-hospital patient self-management education and symptom monitoring may enable persons with SCI to better manage their health following discharge and prevent unnecessary secondary medical complications.

Relationships with peers, family and staff influenced the participant's motivation toward rehabilitation and recovery. Participants felt frustrated by the perceived discouraging demeanor of some staff, or not feeling sufficiently challenged during structured rehabilitation programs. Conversely, building relationships over time with staff created a positive dynamic that encouraged participation in their recovery. These findings align with a qualitative meta-analysis from 2007, which identified that the SCI patient experience of rehabilitation was largely influenced by the qualities of the staff at enhancing patient self-esteem (14).

Study participants often perceived limited provider knowledge of SCI in the acute care setting as well as inconsistencies in SCI care practices between hospitals. Some participants noted significant differences in care practices related to bladder and skin care, which often left them unsure of best practices and how to manage their own care at home. The low prevalence of SCI can challenge non-specialist care providers within and outside the hospital setting on staying up-to-date on current best practices (34). Opportunities to enhance care provider knowledge could include continuing education, mentoring, updated best practice guidelines, and enhanced communication between specialist and non-specialist care providers (34).

The complexity and diversity of SCI treatment and management strategies warrants the need for multidisciplinary collaboration and coordination to ensure providers have the resources and tools to consistently support patient care. One study used a step-wise multidisciplinary team approach in the acute care setting for traumatic SCI to implement a dedicated SCI service. This service involved bi-weekly SCI sessions that included: collaboration of surgical specialists and allied health care professionals; developing and implementing SCI bundle order sets to promote standardization of care, coordination of surgical services; patient and family education; and promoting social lifestyle changes at discharge (35). These type of approaches may enhance continuity of care and standardized care practices for patients across diverse care settings. Implementing SCI standards of care provides an opportunity to re-design in-hospital SCI care provision, reduce unnecessary complications following discharge, improve the patient experience, and address health issues that lead to frequent re-hospitalization (36, 37). However, identifying optimal management strategies for SCI is challenging (38). There is limited rehabilitation research, inconsistent outcome measures, and heterogeneous populations to support SCI best practice standards (1). Furthermore SCI rehabilitation is complex, often involving multiple treatments by multiple care providers and input from the patient and their care providers. Patient participation in rehabilitation is considered a cornerstone of SCI care and is strongly encouraged to promote patient involvement in care planning and decision-making (19). Developing and implementing standardized outcome measures of SCI care that are both evidence-informed and patient-centered can enable health systems to continuously learn from patients and providers to update practice, while enabling comparisons across sites for quality improvement (39). A 2013 publication from the Institute of Medicine recommended healthcare systems adopt a continuous learning health system to support the development of standardized clinical processes and team-based care (40). Learning health systems use measurement to inform practice and practice to inform evidence and quality improvement of care (40). In Canada, the Rick Hansen Spinal Cord Injury Registry collects SCI patient data during the in-hospital phase of care as well as through community follow-up for years following injury (41). These types of registries can enable researchers to investigate the relationship between indicators of SCI inpatient care, patient outcome measures, and long-term patient outcomes to identify areas of health system improvement.

### Need for Effective Communication

Poor communication with healthcare providers emerged as a significant negative experience among individuals with SCI. These negative experiences are addressed within the Canadian Patient's Bill of Rights, including receiving appropriate and timely care, being treated with dignity and respect, and receiving information relating to proposed treatment and options (42). Participants felt some providers lacked empathy toward their condition, and noted situations where they felt they were not treated appropriately; did not receive timely information about their care; and were not included in their care decision making. Many of these negative interactions were found to reduce overall trust in the providers and was perceived to hinder their ability to achieve their desired rehabilitation outcomes. This aligns with findings from a qualitative study on the meaning, process, and consequences of care during SCI rehabilitation, which found that patients who perceived care providers as non-caring felt more hindered to successfully attain their desired rehabilitation outcomes (15). In the present study, many participants expressed that some care providers, particularly during the acute care phase, were unsympathetic of their condition and generally de-motivating when discussing their prognosis and recovery trajectory.

A common theme among participants was unmet expectations of inpatient rehabilitation. This finding is similar to results by Garrino et al. who found that patients with SCI expected greater treatment and rehabilitation care and less time in a clinical or medical setting compared to an acute care hospital (43). Patient expectations can refer to many areas including general expectations about health care provision, health care provider's interpersonal and clinical skills, or receiving information about their care (44). In general, patient needs for support and information are found to be more valued than technical interventions from the patient perspective (44, 45). Our findings demonstrate that, prior to transition to inpatient rehabilitation, patients may benefit from education about what to expect during their stay in inpatient rehabilitation, as well as clear discussions on patient and provider expectations of care. This may also apply to providing appropriate education to patients to prepare for discharge, return to home, and community re-integration.

### Navigating Appropriate Care Supports

Navigating care supports, including identifying and accessing government funding opportunities, as well as peer and community resources, were useful in supporting participants throughout their care journey, especially during discharge and transition to home or assisted living facility. A contributing factor to quality life following an acquired disability is making connections with others, often through participation in the community (46). A qualitative study of persons with SCI identified strong social supports as an important facilitator for participation and connection with community (46). They recommend family and close friends be involved in the SCI rehabilitation process, and be engaged in the facilitation of social and community activities as early as possible (14, 46). This finding was also observed in the present study, where most participants had a family member support their transition home and way finding of community resources and supports.

The importance of peer support workers has been recommended to model a positive future for persons that recently acquired an SCI (46–48). In the present study, several participants recommended peer-support volunteers. These volunteers provided in-hospital education and community supports for persons with SCI. Many participants appreciated peer support as providing information on community resources and modeled positive life experiences outside of the hospital. However, study participants that received SCI care during the COVID-19 pandemic noted limited or no access to in-hospital peer support due to public health restrictions. This was troublesome for participants of older age that struggled to connect with peers through virtual means on their own. Although this was not the primary aim of this study, this warrants a need for alternative strategies to provide in-hospital peer support. Advances in virtual health can provide opportunities to connect with patients in-hospital during restrictions such as COVID-19 (49). Emerging technologies have been proposed to support the facilitation of virtual peer support in rehabilitation care settings (50). This can include webinars, video phone calls, chatbots, and virtual reality programs, which could enable virtual peer support or contact with family and friends. They can also be used to deliver emotional support to enhance patient motivation (51). Peer support, whether in-person or virtual, should be embedded in patient care plans as early as acute care to best support preparedness for discharge and transition to community.

### Strengths and Limitations

This study explored in-depth the experience of persons with SCI regarding in-hospital care from acute care to inpatient rehabilitation. Key strengths of the methodology include the diversity of participants, in-depth interviews, and two independent coders for all transcripts, which strengthens the rigor of our findings and supports ensure transferability. A notable limitation is the eligibility criteria for participation. Participants were eligible up to 10 years post-injury, which may result in recall bias since their initial hospital experience. Additionally, most participants were biologically male, which may limit the interpretations of the findings to the female experience. Of the ten participants, three received initial care outside of the province, which may influence their overall perceptions of care. Due to restrictions of the COVID-19 pandemic, all interviews were conducted over virtual platforms or telephone, which may reduce participant accessibility to participate, and comfort with the interviewer.

## Conclusion

The experience of initial hospital care for persons with SCI is complex and has many barriers. Self-empowerment, motivation from staff, appropriate informational needs, peer and family support, and resource way finding can help better support patients to overcome these barriers and may enhance quality of life post-discharge. These findings can inform recommendations to better incorporate community supports and discharge planning to enhance rehabilitation stay and improve the transition to back to community.

## Data Availability Statement

The dataset includes transcripts with identifiable patient data. Representative quotes can be found in Table 3 of the manuscript. Requests to access the datasets should be directed to jacqueline.krysa@albertahealthservices.ca.

## Ethics Statement

This study involved human participants and was reviewed and approved by University of Alberta Research Ethics Board. The patients/participants provided their written informed consent to participate in this study.

## Author Contributions

JK, KP, EP, and CH led the conception and design of the study. JK performed the interviews and wrote the manuscript. MG transcribed all interviews. JK and MG extracted and analyzed transcripts for emerging themes. KP, CH, EP, and RM reviewed and edited the manuscript for clarity. All authors have contributed to the manuscript in accordance with the criteria for authorship.

## Funding

JK was funded through the Alberta Paraplegic Foundation. CH is a Canadian Institutes for Health Research Principal Investigator. MG was funded through the Faculty of Nursing Studentship from the University of Alberta.

## Conflict of Interest

The authors declare that the research was conducted in the absence of any commercial or financial relationships that could be construed as a potential conflict of interest.

## Publisher's Note

All claims expressed in this article are solely those of the authors and do not necessarily represent those of their affiliated organizations, or those of the publisher, the editors and the reviewers. Any product that may be evaluated in this article, or claim that may be made by its manufacturer, is not guaranteed or endorsed by the publisher.
