# Supplementary material for: Empowerment, Communication, and Navigating Care: The Experience of Persons With Spinal Cord Injury From Acute Hospitalization to Inpatient Rehabilitation
Source: Front Rehabil Sci. 2022 May 31;3:904716. doi: 10.3389/fresc.2022.904716 (PMC9397833; doi:10.3389/fresc.2022.904716)
Supplement: Supplementary file 1 [file Data_Sheet_1.docx]

Semi-Structured Interview Guide

Title of Study: Experience of Patients with Acute Spinal Cord Injury (SCI): From Acute Hospitalization to Inpatient Rehabilitation

1. Interview Introduction

Thank you for taking the time to share your care experiences with us. As noted in the informed consent form, your participation is entirely voluntary. You do not have to answer any question if you do not want to, and we can stop at any time.

We expect that this interview will take about 30-60 minutes. If you would like to take a break for any reason during the interview, please let us know.

This interview will be recorded so that we can take notes of your answers, however, your name and any other personal information will remain confidential. When we share it within the rest of our team and beyond that it’s at de-identified quotes or kind of the high-level main ideas that people share. Is this alright with you?

Do you have any questions or concerns before we begin?

We are first going to ask a few questions about you:

1. What is your preferred identity (e.g., male or female)?
2. What is your age?

2. Interview Guide

We want to learn more about the care experiences of patients and care givers with a spinal cord injury during their initial acute hospital and inpatient rehabilitation. We hope to use your experiences to improve and standardize care for patients who are hospitalized with a spinal cord injury in the future.

1. How long ago did your initial spinal cord injury occur?
2. How did your initial spinal cord injury happen?
3. Where did you receive your initial care for your spinal cord injury?
4. How did you feel about the quality of your acute care for spinal cord injury?
5. Following initial acute hospital care, most patients with spinal cord injuries are transferred to different units such as inpatient rehabilitation. How did you feel about the quality of your inpatient rehabilitation care for spinal cord injury?

[*Based on content discussed, some or all of the following specific questions will be asked.]*

1. What were your realistic expectations of hospital and rehabilitative post-injury and where did you get information for these expectations?
2. Did you receive adequate emotional/psychological support in hospital after your injury?
3. Did your significant other / family receive adequate emotional/psychological support after your injury?
4. Did you feel you and your family were included in your care decisions early during your stay in the acute hospital setting?
5. If you had pain, did you feel your pain was well managed in the hospital?
6. With respect to bowel and bladder care; do you feel you received adequate education on management during your hospital stay?
7. With respect to skin care, do you feel you received appropriate education on management during your hospital stay?
8. How did you find the consistency of your care?
9. How did you feel about the transition from acute, hospital care to inpatient rehabilitation?
10. Did you feel you and your family were well prepared for your transition back to the community?
11. Is there anything else you can tell us about your stay that you think could improve care quality for patients with spinal cord injuries?
